# Risk factor analysis and prediction model construction for delayed erythroid hematopoietic reconstitution after allogeneic hematopoietic stem cell transplantation: a single-center retrospective study

**DOI:** 10.3389/fonc.2026.1862415

**Published:** 2026-07-13

**Authors:** Qianqian Zhang, Fangfang Ge, Lijie Han, Mengmeng Yan, Rong Guo, Zhongxing Jiang

**Affiliations:** Department of Hematology, The First Affiliated Hospital of Zhengzhou University, Zhengzhou, China

**Keywords:** allogeneic hematopoietic stem cell transplantation, delayed erythroid hematopoietic reconstitution, logistic multivariate analysis, prediction model, risk factors

## Abstract

**Background:**

Allogeneic hematopoietic stem cell transplantation (allo-HSCT) represents a potential therapeutic option for various hematological disorders. With continuous advancement in transplantation techniques and the expansion of indications, clinical issues associated with delayed erythroid hematopoietic reconstitution (DEHR) have become increasingly prominent.

**Methods:**

This retrospective study identified independent risk factors through univariate and multivariate logistic regression analyses, subsequently constructing a prediction model. Model discrimination, calibration, and clinical utility were assessed using the area under the curve (AUC), calibration curves, Hosmer-Lemeshow tests, and decision curve analysis. Overall model performance was evaluated via Nagelkerke R², with internal validation conducted using the bootstrap method.

**Results:**

Logistic multivariate analysis results showed that the ferritin level before transplantation (OR = 1.001, 95% CI 1.000-1.001), the severity of anemia before transplantation (OR = 3.740, 95% CI 1.115-12.541), and whether the ABO blood type of the donor and recipient were compatible (OR = 3.283, 95% CI 1.262-8.540) were independent risk factors (*P* < 0.05). And the model’s AUC was 0.835 (95% CI: 0.759–0.910), with the slope of the calibration curve approaching 1, the H-L test *P* = 0.346, and Nagelkerke R² = 0.349.

**Conclusion:**

The nomogram constructed based on pre-transplant ferritin levels, pre-transplant anemia severity, and donor-recipient ABO blood group compatibility demonstrates high predictive value for the occurrence of DEHR after allo-HSCT. This provides a reference for the early identification of high-risk individuals.

## Introduction

Allo-HSCT constitutes a vital therapeutic modality for numerous malignant and nonmalignant hematological disorders (1). Successful post-transplantation hematopoietic reconstitution, particularly the rapid engraftment of neutrophils and platelets, is crucial to mitigate the risk of early infections and hemorrhage while ensuring transplant success. Consequently, this has emerged as a focal point of clinical attention and prior research (2–4). In contrast, the rules and imaging factors of DEHR after allo-HSCT have received less attention, and related research is limited. This situation primarily stems from several factors. First, DEHR typically manifests as a relatively slow, progressive process that lacks the clinical urgency directly triggered by neutropenia or severe thrombocytopenia. Second, the absence of unified, clear assessment criteria and study endpoints for erythroid reconstitution, coupled with the relatively low occurrence rate of classic complications such as pure red cell aplasia (PRCA) post-transplantation, poses challenges for conducting large-scale clinical research. In addition, the mechanisms underlying DEHR are complex, representing a comprehensive interplay of multidimensional abnormalities in post-transplantation immunity, microenvironment, progenitor cell function, and regulatory networks. Finally, routine blood transfusion support can correct anemia to a certain extent, objectively diminishing the perceived urgency to address the fundamental issue of erythroid reconstitution. However, with the continual advancement of allo-HSCT techniques and expansion of indications, particularly the widespread application of haploidentical and blood-type-mismatched transplants, clinical issues related to DEHR have become increasingly prominent and are now receiving greater attention (5, 6). Against this backdrop, systematically investigating the risk factors for DEHR after allo-HSCT and developing effective risk prediction tools are of significant clinical importance. This approach facilitates the early identification of high-risk individuals, guides targeted interventions, and ultimately improves long-term patient survival outcomes. Therefore, this study was designed to clarify the independent risk factors for DEHR after allo-HSCT by retrospectively analyzing single-center data and constructing a visual individualized prediction model to supply a practical reference for clinical decision-making.

## Materials and methods

### Study design and patient selection

In this research, we consecutively enrolled 165 patients who underwent allo-HSCT at the Hematopoietic Stem Cell Transplantation Center of the South Campus of the First Affiliated Hospital of Zhengzhou University from June 2023 to December 2024 as research subjects. Based on the assessment of erythroid hematopoietic reconstitution after allo-HSCT, 165 patients were categorized into the non-delayed erythroid hematopoietic reconstitution group (NEHR group, n=132) and the delayed erythroid hematopoietic reconstitution group (DEHR group, n=33) (**Table 1**). Compliance with the Helsinki Declaration was maintained throughout this study, which was granted approval by the Ethics Committee of The First Affiliated Hospital of Zhengzhou University. Owing to the retrospective design, obtaining informed consent was deemed unnecessary. Patient clinical data were anonymized and de-identified before study initiation to ensure sufficient protection of patient privacy.

**Table 1:** Comparison of demographic data, clinical characteristics, and transplantation profiles between NEHR and DEHR groups.

Characteristics	Total	NEHR	DEHR	*P*
	N = 165	N = 132	N = 33	
Sex (%)				0.095
male	104 (63.0)	79 (59.8)	25 (75.8)	
female	61 (37.0)	53 (40.2)	8 (24.2)	
Age (y)		33 (19,48)	34 (24,49)	0.428
Disease (%)				0.005
AA+ MDS	73 (44.2)	51 (38.6)	22 (61.4)	
AML+ALL+ lymphoma+ others	92 (55.8)	81 (61.4)	11 (38.6)	
Complication (%)				0.907
no	144 (87.3)	115 (87.1)	29 (87.9)	
yes	21 (12.7)	17 (12.9)	4 (12.1)	
Fibrosis (%)				0.933
no	114 (69.1)	91 (68.9)	23 (69.7)	
yes	51 (30.9)	41 (31.1)	10 (30.3)	
Chromosome (%)				0.736
normal	114 (69.1)	92 (69.7)	22 (66.7)	
abnormal	51 (30.9)	40 (30.3)	11 (33.3)	
Time (d)		150 (93,300)	170 (75,450)	0.224
Anemia (%)				0.001
normal-mild	77 (46.7)	71 (53.8)	6 (18.2)	
moderate-severe	88 (53.3)	61 (46.2)	27 (81.8)	
Ferritin (ng/ml)		1241 (6077.7,1542.1)	1697 (1358.0,3044.5)	0.000
Pretreatment (%)				1.000
BUCY-based	89 (53.9)	70 (53.0)	19 (57.6)	
TBI-based	22 (13.3)	20 (15.2)	2 (6.06)	
others	54 (32.7)	42 (31.8)	12 (36.4)	
Pre-GVHD (%)				0.097
CsA+MTX+MMF	26 (15.8)	23 (17.4)	3 (9.09)	
CsA+MTX+MMF+ATG	70 (42.4)	57 (43.2)	13 (39.4)	
CsA+MTX+MMF+ATG+CD25	61 (37.0)	47 (35.6)	14 (42.4)	
others	8 (4.85)	5 (3.79)	3 (9.09)	
HLA (%)				0.368
HLA Mismatch ≤1	56 (33.9)	47 (35.6)	9 (27.3)	
HLA Mismatch ≥2	109 (66.1)	85 (64.4)	24 (72.7)	
PRA (%)				0.497
negative-weakly positive	132 (80.0)	107 (81.1)	25 (75.8)	
strongly positive	33 (20.0)	25 (18.9)	8 (24.2)	
Doner type (%)				0.627
related donor	146 (88.5)	116 (87.9)	30 (90.9)	
unrelated donor	19 (11.5)	16 (12.1)	3 (9.1)	
Gender consistency (%)				0.240
conformity	95 (57.6)	79 (59.8)	16 (48.5)	
inconformity	70 (42.4)	53 (40.2)	17 (51.5)	
Blood type (%)				0.010
conformity	89 (53.9)	78 (59.1)	11 (33.3)	
inconformity	76 (46.1)	54 (40.9)	22 (66.7)	
Source of stem cell (%)				0.386
PB	147 (89.1)	119 (90.2)	28 (84.8)	
PB+UCB	18 (10.9)	13 (9.85)	5 (15.2)	
CD34^+^ cell infusion (%)				0.910
<5	46 (27.9)	36 (27.3)	10 (30.3)	
[5,10]	85 (51.5)	70 (53.0)	15 (45.5)	
>10	34 (20.6)	26 (19.7)	8 (24.2)	
MNC infusion (%)				0.813
<10	68 (41.2)	55 (41.7)	13 (39.4)	
≥10	97 (58.8)	77 (58.3)	20 (60.6)	
MSC infusion (%)				0.701
no	131 (79.4)	104 (78.8)	27 (81.8)	
yes	34 (20.6)	28 (21.2)	6 (18.2)	
EBV infection (%)				0.324
no	163 (98.8)	131 (99.2)	32 (97.0)	
yes	2 (1.21)	1 (0.76)	1 (3.03)	
CMV infection (%)				0.313
no	156 (94.5)	126 (95.5)	30 (90.9)	
yes	9 (5.45)	6 (4.55)	3 (9.1)	
aGVHD (%)				0.001
no	98 (59.4)	87 (65.9)	11 (33.3)	
yes	67 (40.6)	45 (34.1)	22 (66.7)	

BUCY-based: Busulfan + Cyclophosphamide-based; TBI-based: Total Body Irradiation-based; CsA: Cyclosporine A; MTX: Methotrexate; MMF: Mycophenolate Mofetil; ATG: Antithymocyte Globulin; CD25: Anti-CD25 Monoclonal Antibody; PB: peripheral blood; PB+UCB: peripheral blood combined with umbilical cord blood.

The inclusion criteria were as follows: diagnosis of malignant or non-malignant hematological diseases, such as aplastic anemia (AA), acute myelocytic leukemia (AML), acute lymphoblastic leukemia (ALL), myelodysplastic syndrome (MDS), and others according to the WHO diagnostic criteria (2016 version); HSCT was performed for the first time with no prior hematopoietic stem cell or other organ transplantations; the post-transplantation survival period was at least 1 month; and the clinical and laboratory examination data of patients were complete.

The exclusion criteria were as follows: autologous hematopoietic stem cell transplantation (auto-HSCT); patients with missing clinical data or laboratory records; and patients or donors with concomitant significant organ dysfunction (e.g., cardiac, hepatic, or renal) or psychiatric disorders.

The following conditions must be met approximately 30 days after hematopoietic stem cell reinfusion: 1) Hemoglobin level < 70 g/L, without red cell transfusion; 2) Genetic testing confirming complete hematopoietic system chimerism from donor cell; 3) No recurrence of the primary disease; 4) No confirmed bleeding events. Based on these criteria, patients were categorized into NEHR and DEHR groups.

### Data collection

The clinical information gathered for this research encompasses the following three aspects:

1. Basic information, including sex, age at transplantation, diagnosis of the primary disease, and presence of underlying comorbidities.
2. Treatment history and pre-transplant status:
Disease characteristics, including the degree of myelofibrosis at diagnosis and chromosomal status at diagnosis.
Pre-transplant status and conditioning regimen: Time from initial diagnosis to transplantation, severity of anemia before transplantation, pre-transplant ferritin levels, pre-transplant conditioning regimen, GVHD prophylaxis regimen, and panel reactive antibody (PRA) status.
3. Transplant-related factors and post-transplant events: Donor and graft characteristics, including donor type (related/unrelated), human leukocyte antigen (HLA) compatibility, donor-recipient ABO blood group compatibility and sex matching, the source of the graft, infused CD34+ cell dose and mononuclear cell (MNC) dose, and whether mesenchymal stem cells (MSC) were combined with the infusion.

Early complications after transplantation: document the incidence of Epstein-Barr Virus (EBV) infection, Human Cytomegalovirus (CMV) infection, and acute graft-versus-host disease (aGVHD) within 30 days post-transplant.

### Statistical analysis

First, we employed univariate assessment to screen for potential risk factors, with variables yielding *P* < 0.05 being included in the subsequent multivariate analysis, which was conducted to identify the independent risk factors for DEHR following allo-HSCT. Based on the aforementioned identified risk factors, we utilized R version 4.5.0 to construct a nomogram model predicting an individual’s risk of DEHR. To evaluate the model’s performance, the following validation procedures were conducted: the model’s discriminatory capability was assessed by plotting the Receiver Operating Characteristic (ROC) curve and calculating the AUC; the consistency between predicted probabilities and actual observed incidence rates was evaluated using calibration curves; clinical utility was analyzed using decision curves and the Hosmer-Lemeshow goodness-of-fit test (H-L test). The overall model performance was evaluated using Nagelkerke R^2^, and the model was validated through bootstrap resampling.

## Results

### Baseline data comparison

In the current study, 165 patients that met all inclusion requirements were incorporated, among whom 33 (20.0%) experienced DEHR and 132 (80.0%) did not. With regard to sex, no statistically significant disparities existed between the two groups. In addition to this, there are also factors such as chromosomal karyotype, degree of myelofibrosis, transplant type (related/unrelated), donor-recipient sex matching, HLA locus compatibility, PRA, source of the graft, conditioning regimen, infused CD34+ cell dose and MNC dose, whether combined with MSC transfusion, BKV infection, and CMV infection within 30 days after transplantation (*P* > 0.05). In contrast, the two groups differed significantly (*P* < 0.05) with respect to the following baseline features: primary disease, pre-transplant ferritin levels, pre-transplant anemia severity, donor-recipient ABO blood group compatibility, and incidence of aGVHD within 30 days post-transplantation (**Table 1**).

Analysis of risk factors for DEHR after allo-HSCT: statistically significant variables (*P* < 0.05) from univariate analysis were entered into a multivariable logistic regression model. The results indicated that elevated pre-transplant ferritin levels, moderate or severe anemia prior to transplantation, and donor-recipient ABO blood group compatibility were independent risk factors for DEHR after allo-HSCT (all *P* < 0.05). The specific assignments, odds ratios (OR), and 95% confidence intervals (95% CI) for all factors are detailed in the table below (**Table 2**).

**Table 2:** Multivariate analysis of factors influencing erythroid hematopoietic reconstitution after allo-HSCT.

Variate	B	OR	95% CI	*P*
disease	0.345	1.411	0.490-4.066	0.523
anemia	1.319	3.740	1.115-12.541	0.033
ferritin	0.001	1.001	1.000-1.001	0.006
blood type	1.189	3.283	1.262-8.540	0.015
aGVHD	0.903	2.467	0.951-6.396	0.063

### Risk-stratified model establishment

**Figure 1:**
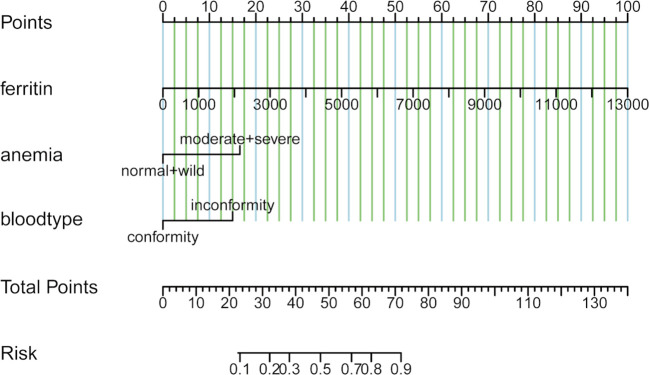
Nomogram. Nomogram for predicting DEHR after allo-HSCT.

Given the three independent risk factors noted above, a simple risk scoring system was established to facilitate clinical application. This model converts the value of each variable into a score, enabling the probability of delayed events occurring in each patient to be estimated intuitively by correlating the total score with the corresponding risk axis (**Figure 1**).

### Validation and evaluation of the nomogram prediction model

**Figure 2:**
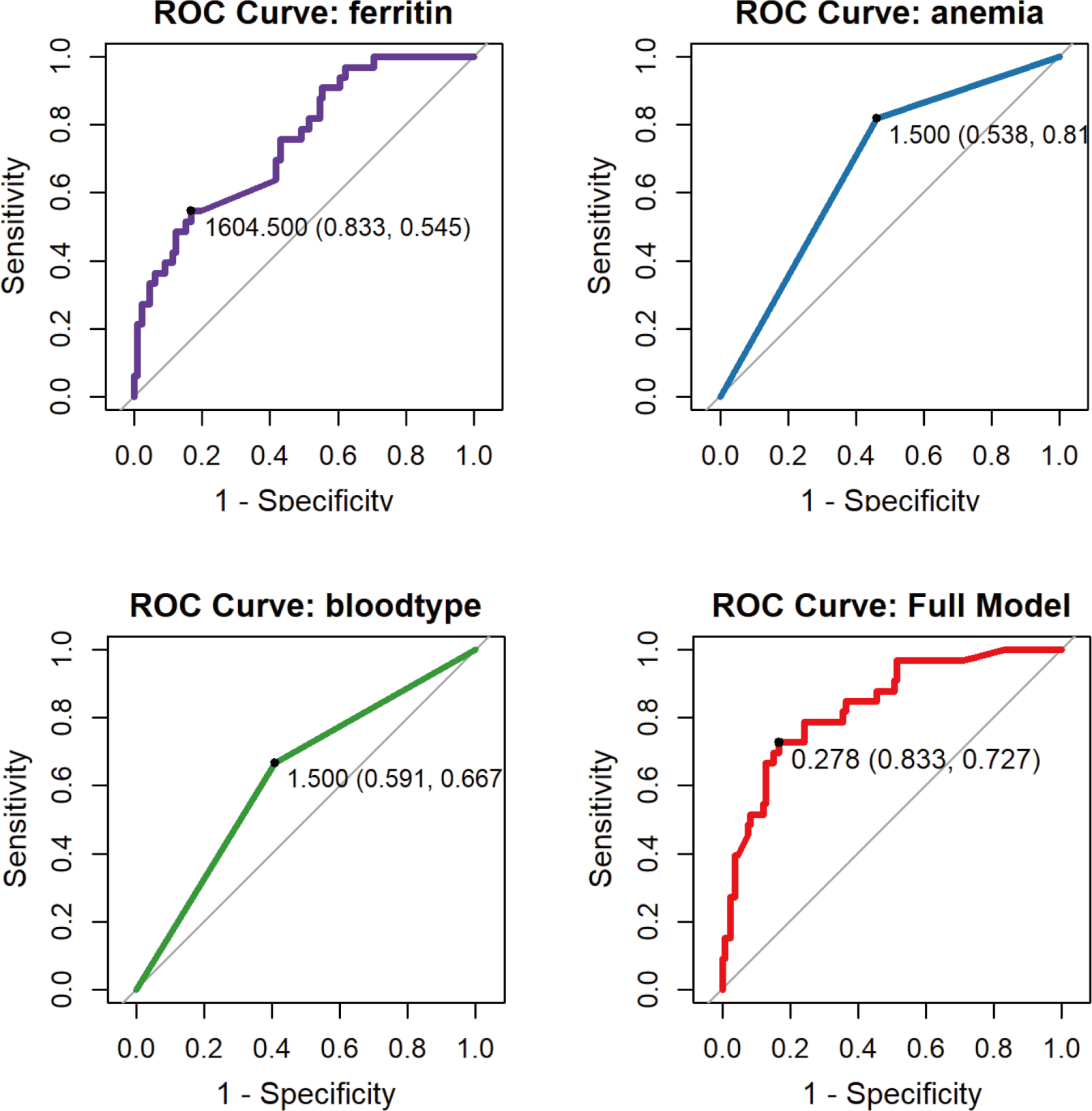
Individual ROC curves for predictive variables and full model. This panel of receiver operating characteristic (ROC) curves displays the discriminative ability of individual variables (ferritin, anemia, bloodtype) and the full prediction model for DEHR after allo-HSCT. Each subplot corresponds to one predictor, with curves illustrating the trade-off between sensitivity (true positive rate) and 1-specificity (false positive rate) for predicting the outcome.

**Figure 3:**
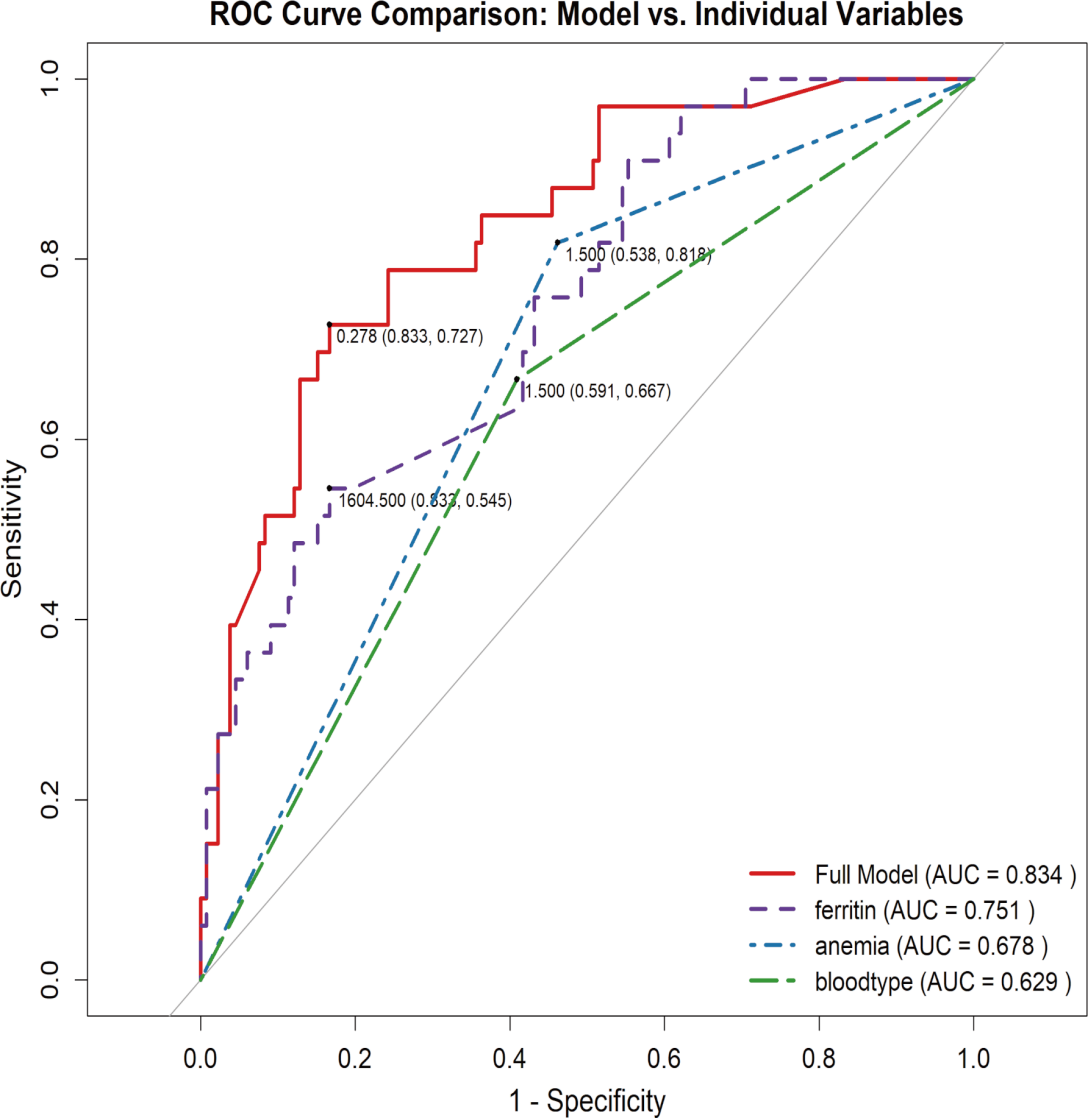
ROC curve comparison: full model vs. individual variables. This ROC curve plot compares the discriminative performance of the full prediction model and individual variables (ferritin, anemia, bloodtype) for DEHR after allo-HSCT. The full model (AUC = 0.834) demonstrates superior discriminative ability relative to individual variables (ferritin: 0.751; anemia: 0.678; bloodtype: 0.629).

We performed a comprehensive validation to evaluate the predictive capability of the proposed model. Firstly, the discrimination of the model was evaluated by plotting ROC curve analysis. The results indicated an AUC of 0.835 (95% CI: 0.759–0.910) for predicting DEHR, demonstrating its sound discriminatory capacity (**Figures 2**, **3**).

**Figure 4:**
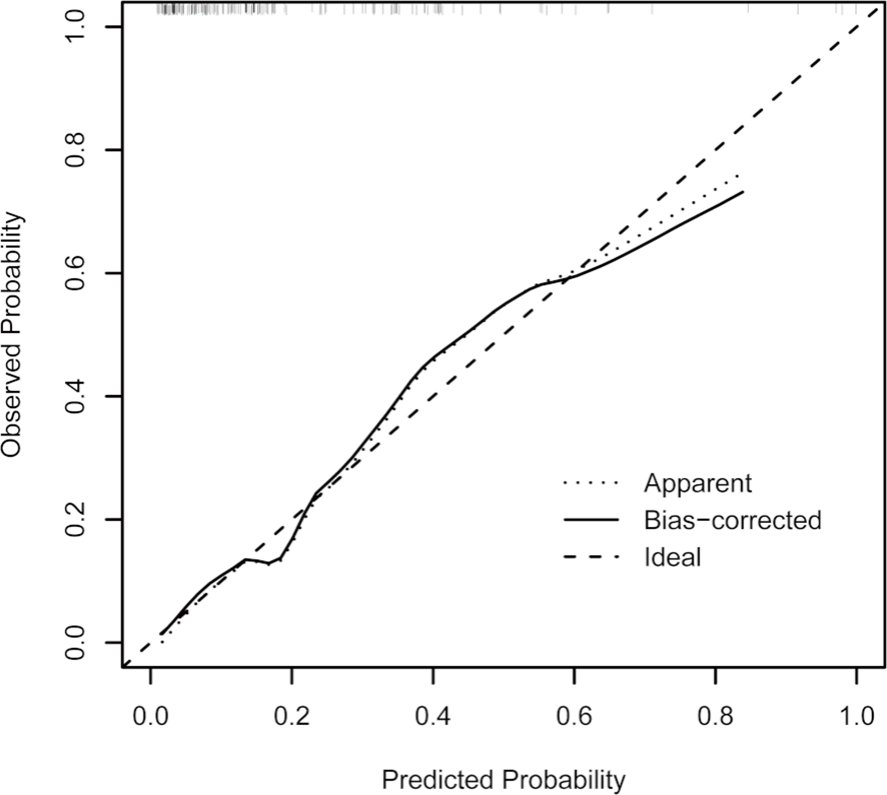
Calibration curve. This calibration curve evaluates the agreement between predicted probabilities (x-axis) and observed frequencies (y-axis) of DEHR after allo-HSCT. The bias-corrected curve closely aligns with the ideal line (slope near 1), indicating good calibration of the model.

**Figure 5:**
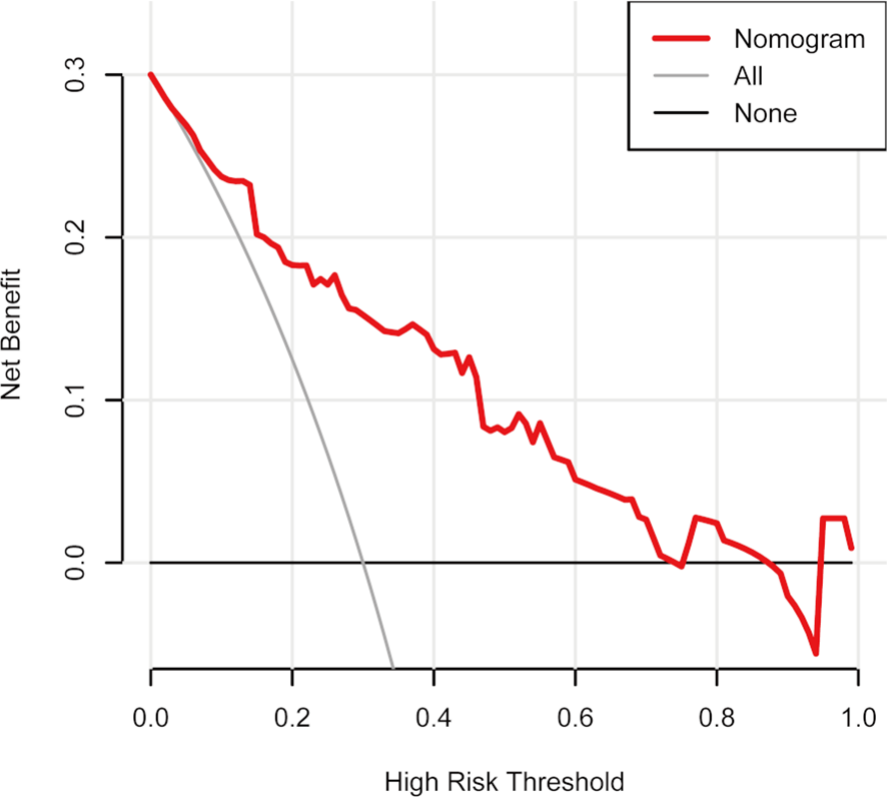
Decision curve analysis. This decision curve evaluates the clinical utility of the nomogram by quantifying *Net Benefit* across a range of high-risk thresholds for DEHR after allo-HSCT. Curves compare the nomogram, “All” (intervening for all patients), and “None” (intervening for no patients).

For the internal validation of the model, the bootstrap method was employed to assess its stability and discriminative ability. After 200 resampling iterations, the adjusted AUC reached 0.828, with kappa = 0.318, indicating the robust predictive performance of the model. Concurrently, the calibration curve demonstrated good consistency between the predicted probabilities and actual observed frequencies, with a slope approaching 1. The H-L test yielded χ² = 0.889, *P* = 0.346, confirming satisfactory model calibration (**Figure 4**). Finally, decision curve analysis demonstrated that across a broad scope of probability thresholds, the clinical net benefit rate achievable using this prediction model consistently exceeded that of the two extreme strategies—”all interventions” and “no interventions”—thereby validating the model’s strong clinical utility (**Figure 5**).

**Figure 6:**
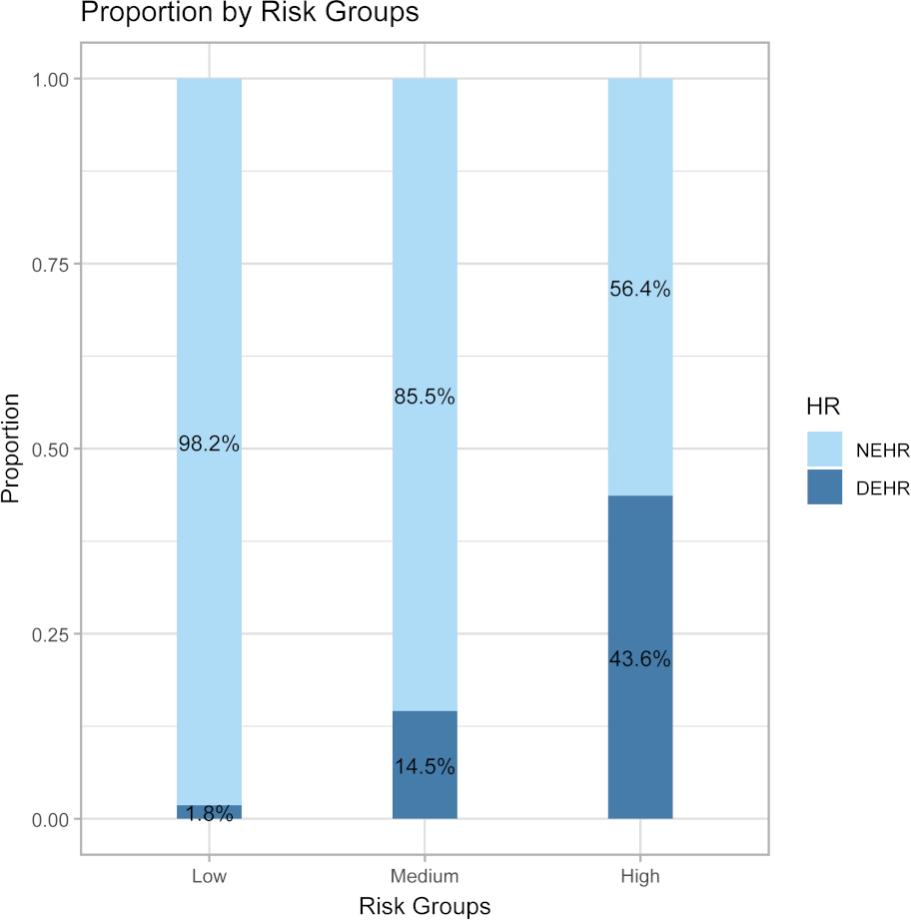
Proportion of erythroid hematopoietic reconstitution by risk groups.

Via the model’s predict function, we computed individual total scores for each patient, which stratify them into three risk groups: low-risk, intermediate-risk, and high-risk. Within these three groups, the percentage of patients experiencing DEHR progressively increased with increasing risk stratification (**Figure 6**).

## Discussion

In this single-center retrospective analysis, we preliminarily investigated the factors associated with DEHR after allo-HSCT. The prediction model constructed based on these factors demonstrated discriminatory capability for internal validation, providing a reference for subsequent research in this field. Through univariate and multivariate logistic regression analyses, this study demonstrated that pre-transplant ferritin levels constitute an independent risk factor for DEHR after allo-HSCT. This finding is consistent with the established theory that iron overload may lead to erythropoietic dysfunction (7–9). Serum ferritin is an acute-phase reactant protein that is elevated during systemic inflammatory responses (10). Iron overload may manifest to some extent as elevated ferritin levels, and pre-transplant ferritin levels have been shown to correlate with long-term post-transplant outcomes (11–15). With respect to DEHR after allo-HSCT, our findings align with those of Malki et al., who identified “ferritin-related risk factors” for delayed platelet engraftment, supporting the broad inhibitory effect of iron overload on multilineage hematopoiesis. This observation suggests that pre-transplant assessment and necessary interventions for patients at risk of iron overload may improve post-transplant hematopoietic recovery (16). Iron overload inhibits BFU-E colony formation and erythroid differentiation in rat and human bone marrow progenitor cells *in vitro*. Cells exposed to excessive iron exhibit dysplastic changes, increased intracellular reactive oxygen species levels, and reduced expression of anti-apoptotic genes (17). Experiments by Wenbin An et al. demonstrated increased gene expression involved in iron uptake, sensing, and transport in stem and progenitor cells from patients with myeloproliferative syndrome and established erythrocyte-specific iron metabolism as a novel potential therapeutic target for reversing ineffective myeloproliferation (7).

Whether to initiate treatment for iron overload and the optimal timing for such interventions remain the subjects of ongoing debate. Experiments conducted by Wenbin An et al. in mice confirmed that iron chelators could improve ineffective erythropoiesis in mice with myelodysplastic syndromes (7). Trials have demonstrated that patients with low-risk myeloproliferative disorders undergoing iron chelation therapy exhibit prolonged event-free survival (18). Zeng et al. indicated that iron chelation therapy effectively improved hematopoietic function among hematological malignancy patients who experienced iron overload after allo-HSCT, with favorable safety profiles (19). Studies by Pan TZ et al. demonstrate that pre-transplant iron overload may affect total survival rate and transplantation-related mortality in patients with serum albumin-associated anemia after allo-HSCT (20).

However, current research in this field has the following shortcomings: the singularity of iron metabolism indicators, which solely measure ferritin without integrating transferrin, serum iron, or ferritin saturation, making it difficult to comprehensively reveal the dynamic requirements of erythroid hematopoiesis for iron metabolism, together with the lack of cell line-specific mechanisms. Subsequent investigations should focus on cell line-specific mechanisms and intervention strategies for iron metabolism. By integrating transcriptomics and metabolomics technologies, changes in the gene expression profiles of erythroid progenitor cells under high-iron conditions can be identified, thereby enabling the identification of precise therapeutic targets to improve delayed erythroid reconstitution post-transplantation. Additionally, multicenter prospective studies should be implemented to explore the specific efficacy of iron chelation therapy for erythroid hematopoietic recovery in multiple disease conditions.

The impact of prolonged anemia before transplantation on DEHR after allo-HSCT remains poorly characterized in definitive studies. However, several contributing factors may be considered. First, damage to the bone marrow microenvironment: chronic anemia induces bone marrow hypoxia and increases oxidative stress, compromising the microenvironment essential for hematopoietic stem cell survival and diminishing their proliferative and differentiation capacity. Second, under hypoxic conditions, hematopoietic stem cells’ (HSPCs) self-renewal potential diminishes, reducing the efficiency of direct differentiation into erythroid, myeloid, and megakaryocytic progenitor cells, resulting in inadequate hematopoietic recovery post-transplantation. Third, chronic anemia is frequently accompanied by either iron overload (due to repeated transfusions) or deficiency (due to nutritional anemia), both of which suppress hematopoietic stem cell proliferation and interfere with hematopoietic recovery.

This study reaffirms that ABO blood group incompatibility is a recognized significant risk factor for delayed erythroid reconstitution, primarily through the mechanism whereby residual host blood group antibodies attack donor-derived erythroid progenitor cells (21–23). In clinical practice, heightened vigilance is warranted for patients, with enhanced monitoring of post-transplant blood group antibody titers and parameters related to erythroid reconstitution.

Compared to alterations in other cell lineages, hematopoietic abnormalities represent the most characteristic, earliest-appearing, and persistent core changes throughout the course of both aplastic anemia and myelodysplastic syndromes (7, 24). Consequently, these conditions are grouped according to their underlying pathologies. Although the classification of the primary disease and the occurrence of aGVHD within 30 days post-transplantation demonstrated significance in the univariate analysis, neither entered the final model, possibly due to the limited sample size. Moreover, this was an observational cohort that relied on retrospective data and was therefore susceptible to selection bias and confounding factors (25). The complex relationship between these factors and DEHR warrants further clarification through large-scale studies.

The main goal of this research was to provide clinicians with a preliminary, user-friendly risk prediction assessment tool. The value of the newly constructed cut-off score model lies in its ability to integrate multiple discrete risk factors into a single visualized composite score. This may assist clinicians in readily identifying potentially high-risk patients during routine practice, thereby enabling more individualized monitoring and management.

However, the conclusions and values of the model should be interpreted with caution. In the context of single-center allo-HSCT research, the present study exhibits a number of prominent constraints. First, in the form of a retrospective cohort study, selection bias is inevitable, and its limited sample size may prove insufficient to accurately identify all meaningful predictive variables. Second, although the model demonstrated reasonable performance in the internal validation, its generalizability (i.e., applicability to external cohorts) remains unverified. Thus, it should currently serve only as a reference tool for clinical decision-making, not as definitive evidence. The final establishment and clinical implementation of the model urgently requires external validation and optimization through future multicenter, large-sample prospective studies.

## Data Availability

The raw data supporting the conclusions of this article will be made available by the authors, without undue reservation.
